# Efficacy and Safety of Ultrasound‐Guided Nerve Block Versus Local Anesthesia for Arteriovenous Fistula Angioplasty: A Systematic Review and Meta‐Analysis

**DOI:** 10.1002/hsr2.73000

**Published:** 2026-08-05

**Authors:** Wenshen Pu, Zhihu Chen, Zhangjian Xu, Yi Zhang, Zhidao Wang, Bingjie Wang, Ziming Wan

**Affiliations:** ^1^ Department of Nephrology Baoshan People's Hospital Baoshan China; ^2^ Department of Nephrology Chuxiong Yi Autonomous Prefecture People's Hospital Chuxiong China; ^3^ Department of Nephrology The First Affiliated Hospital of Chongqing Medical University Chongqing China

**Keywords:** arteriovenous fistulas, local anesthesia, meta‐analysis, percutaneous transluminal angioplasty, ultrasound‐guided nerve blocks

## Abstract

**Background and Aims:**

Severe intra‐procedural pain commonly complicates percutaneous transluminal angioplasty (PTA) for dysfunctional arteriovenous fistulas (AVFs), undermining patient cooperation and potentially increasing the need for sedative medications. This systematic review and meta‐analysis aimed to evaluate the analgesic efficacy, safety, and clinical performance of ultrasound‐guided nerve block (UGNB) versus local anesthesia (LA) in adult patients undergoing AVF PTA.

**Methods:**

We searched PubMed, Embase, Scopus, the Cochrane Library, and Web of Science for eligible randomized controlled trials and cohort studies published up to September 1, 2025. The primary outcome was intra‐procedural pain rated using VAS or NRS on a 0−10 scale. The secondary outcome was overall safety, assessed using the available complication‐free outcome. Random‐effects models were used for pooled analyses. Heterogeneity was explored using Galbraith plots and leave‐one‐out analyses. Funnel plots were generated for exploratory visualization because fewer than 10 studies were included. Subgroup analyses were performed according to UGNB subtype, and an exploratory subgroup analysis was additionally conducted by study design.

**Results:**

Four eligible studies, including 664 participants from East Asia, were included in the quantitative synthesis. In the primary pain analysis, UGNB was associated with a marked reduction in intra‐procedural pain relative to LA (SMD −2.85, 95% CI −4.70 to −1.01; *p* < 0.001), although heterogeneity remained extreme (*I* ^2^ = 98.45%). Galbraith and leave‐one‐out analyses identified Wen et al. as the most influential study; however, the direction and statistical significance of the pooled effect remained unchanged after omitting any single study. Subgroup analysis by block type showed a significant analgesic effect for BPB, whereas the SCNB subgroup had a wide confidence interval and very high heterogeneity; no significant between‐subgroup difference was detected. The pooled log risk ratio for the complication‐free outcome was −0.02 (95% CI −0.05 to 0.02; *p* = 0.27), indicating no clinically meaningful difference in overall safety between groups. No major nerve injury, pneumothorax, or persistent motor deficits were reported.

**Conclusion:**

UGNB was associated with superior analgesia without a clear increase in adverse events during AVF PTA. Current evidence does not establish a statistically significant difference between BPB and SCNB, and the SCNB subgroup should be interpreted cautiously because of substantial heterogeneity. Larger multicenter trials are needed to compare block techniques directly and to evaluate recovery, patency, and cost‐effectiveness.

## Key Points

What's known: Pain during AVF angioplasty is common and can disrupt procedural workflow.

What's new: Compared with local anesthesia, ultrasound‐guided nerve blocks were associated with markedly lower intra‐procedural pain without a clear increase in adverse events. Subgroup findings did not establish a statistically significant difference between BPB and SCNB.

Clinical implications: Selective adoption of UGNB may improve patient cooperation, reduce the need for rescue sedation, and facilitate same‐day discharge in dialysis access pathways. Technique selection should be individualized according to lesion site, the need for motor preservation, patient tolerance, and local operator expertise.

## 1 Introduction

Chronic kidney disease (CKD) has become a major global health concern, and the number of patients progressing to end‐stage kidney disease (ESKD) continues to rise [1]. Hemodialysis is the most frequently used renal replacement therapy, and establishing and maintaining reliable vascular access are essential for treatment success. Among all access types, the arteriovenous fistula (AVF) is preferred because of its long‐term patency and lower risk of infection [2, 3]. Nevertheless, AVFs are prone to dysfunction, most often caused by stenotic lesions, which remain a leading contributor to access failure [2]. Preserving and restoring AVF patency is therefore crucial for dialysis adequacy and patient outcomes.

Percutaneous transluminal angioplasty (PTA) has become the primary minimally invasive strategy for treating AVF stenosis [4], effectively restoring blood flow and prolonging access survival. However, severe pain during balloon dilation is common. Such pain not only reduces patient cooperation but also necessitates additional sedatives or opioids, which may increase the risk of respiratory depression, hemodynamic instability, or access‐related complications [5]. Since PTA is commonly performed in outpatient or day‐surgery settings, the ideal anesthetic approach should provide reliable analgesia, maintain safety, have a rapid onset, and allow prompt recovery.

Several anesthetic options have been applied in AVF interventions, including general anesthesia (GA), local infiltration anesthesia (LA), intravenous sedation, and regional anesthesia [6, 7, 8]. GA is seldom used due to the high risk of cardiopulmonary complications in uremic patients. LA is convenient, but its effectiveness may be limited by poor drug spread in scarred or stenotic tissue, resulting in inadequate pain relief during balloon inflation [7]. Intravenous sedation can reduce anxiety but carries a risk of respiratory depression and often requires post‐procedural monitoring [5]. In recent years, regional anesthesia, particularly ultrasound‐guided nerve blocks (UGNB), has gained increasing attention.

UGNB techniques—including brachial plexus block (BPB), musculocutaneous nerve (MCN) block, and selective cutaneous blocks of the lateral antebrachial cutaneous nerve (LACN) or superficial radial nerve (SRN)—enable precise sensory blockade of the regions involved in PTA [6, 8]. These techniques may provide effective analgesia, reduce systemic medication requirements, and improve patient comfort and procedural conditions. Comparative evidence regarding UGNB versus LA in AVF PTA remains limited. To address this knowledge gap, we performed a systematic review and meta‐analysis of randomized controlled trials (RCTs) and observational studies comparing the efficacy and safety of UGNB with LA in adult patients undergoing AVF PTA.

## 2 Materials and Methods

This systematic review and meta‐analysis were prospectively registered on PROSPERO (CRD420251250808). The review was conducted and reported in accordance with the Preferred Reporting Items for Systematic Reviews and Meta‐Analyses (PRISMA) 2020 guidelines [9]. Prespecified analyses included the primary pain outcome, overall safety outcomes, and subgroup analysis by UGNB subtype.

### 2.1 Search Strategy

We systematically searched PubMed, Embase, Scopus, the Cochrane Library, and Web of Science from inception to September 1, 2025, using controlled vocabulary and keywords related to “anesthesia,” “hemodialysis,” and “angioplasty.” Gray literature was screened via Google Scholar and conference proceedings, and reference lists of eligible studies and related reviews were hand‐searched.

### 2.2 Eligibility Criteria

We included RCTs and cohort studies enrolling hemodialysis patients undergoing PTA for AVF dysfunction. Interventions included ultrasound‐guided BPB, MCN, or selective cutaneous nerve block (SCNB)—collectively termed UGNB—compared with local infiltration anesthesia (LA). Studies were excluded if they lacked a comparator group, did not report quantitative pain outcomes, or had been retracted.

### 2.3 Study Selection and Data Extraction

Two trained reviewers independently and in duplicate screened titles/abstracts and full texts using pre‐piloted forms and a prespecified workflow. The same reviewers independently extracted data on study characteristics (author, year, design, country, setting, sample size), patient demographics (mean age, sex), intervention/comparator details, outcomes (intra‐procedural pain on visual analog scale [VAS]/numeric rating scale [NRS]; complications), and follow‐up. Disagreements were resolved by consensus or adjudicated by a third reviewer. A PRISMA flow diagram summarizes study selection.

### 2.4 Risk of Bias Assessment

Risk of bias was assessed at the study level using the Cochrane RoB 2 tool for RCTs and the Newcastle–Ottawa Scale (NOS) for observational studies. Judgments were made independently by two reviewers, with third‐party arbitration when required.

### 2.5 Outcomes

The primary outcome was intra‐procedural pain measured on a 0–10 scale using either the VAS or the NRS (also termed NRPS/NPRS). Given the high correlation between VAS and NRS reported in the literature, we treated them as conceptually equivalent and synthesized pain intensity as a single continuous outcome using standardized mean differences (SMDs) [10]. Where necessary, VAS values reported on a 0−100 metric were rescaled to 0−10 prior to pooling. Secondary outcomes included overall safety outcomes, summarized using the available complication‐free data.

### 2.6 Statistical Analysis

All statistical analyses were conducted in accordance with the Cochrane Handbook for Systematic Reviews of Interventions [11] and the improved SAMPL guidelines for statistical reporting in biomedical research [12]. Statistical abbreviations and symbols were defined as follows: SMD, effect size for continuous outcomes; risk ratio (RR), effect size for dichotomous outcomes; 95% confidence interval (95% CI), measure of precision; I^2^ and τ^2^, measures of between‐study heterogeneity; and *α*, the predefined significance level.

Continuous data on intra‐procedural pain were pooled using SMDs with 95% CIs. Cohen's *d* was used as the SMD effect‐size metric in the forest plots. Higher scores on the 0–10 pain scales indicate more severe pain, and a negative SMD represents greater analgesic efficacy of UGNB over LA. Dichotomous safety data were synthesized using log RR with 95% CIs. A continuity correction of 0.5 was applied to datasets with zero event cells. When only standard errors, CIs, or interquartile ranges were reported, standard deviations were calculated using formulas recommended in the Cochrane Handbook [11].

Inter‐study heterogeneity was assessed using Cochran's *Q* statistic, *τ* ^2^, and *I* ^2^. Heterogeneity was considered substantial when *I* ^2^ > 50%. The DerSimonian–Laird random‐effects model was used for analyses with substantial heterogeneity; otherwise, the inverse‐variance fixed‐effect model was used. Heterogeneity was further explored using Galbraith plots and leave‐one‐out influence analyses. Retracted articles were considered ineligible for quantitative synthesis. For the remaining eligible studies, Galbraith‐identified influential studies were not removed from the primary synthesis but were assessed using leave‐one‐out sensitivity analyses.

Classification of analyses was defined as follows. Prespecified analyses included the primary analysis of intra‐procedural pain intensity, analysis of overall safety outcomes, and subgroup analysis comparing BPB and SCNB. Exploratory analyses included Galbraith plots, leave‐one‐out influence analyses, funnel plot visualization, and subgroup analysis by study design. Formal tests for small‐study effects were not emphasized because fewer than 10 studies were included.

All statistical tests were two‐sided, and *p* < 0.05 was considered statistically significant. All quantitative analyses were performed using Stata 18 (StataCorp LLC, College Station, Texas, USA).

## 3 Results

### 3.1 Study Selection

**Figure 1:**
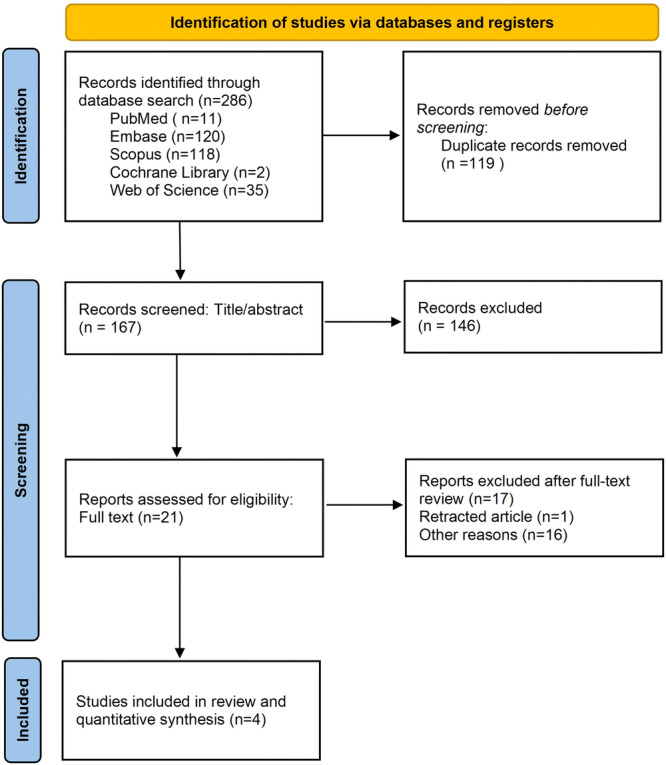
PRISMA 2020 flow diagram of study selection. One otherwise relevant report was excluded during full‐text eligibility assessment and reference validation because it had been retracted. PRISMA, Preferred Reporting Items for Systematic Reviews and Meta‐Analyses.

We identified 286 records from five databases; after removing 119 duplicates, 167 titles and abstracts were screened, and 21 full texts were assessed. During full‐text eligibility assessment and reference validation, one otherwise relevant report was excluded because it had been retracted. Four eligible studies were included in the final quantitative synthesis (Figure 1).

### 3.2 Study Characteristics

Four eligible studies involving 664 participants were included in the quantitative synthesis. These studies were conducted in Korea, Japan, and China. Two were RCTs, and two were retrospective cohort studies. Baseline characteristics, including sample size, age, anesthesia techniques, and pain scales, are summarized in Table 1.

**Table 1:** Summary of included studies and baseline characteristics of their populations.

Study	Design and study arms	Sample size (*n*)	Age, mean ± SD or median (IQR), years	Sex (male/female ratio)	Study period (year)	Intervention (UGNB)	Pain scale
Heo et al. 2020 [13]	RCT (BPB vs. LA)	BPB, 40; LA, 40	BPB, 65.1 ± 12.4; LA, 64.0 ± 11.7	BPB, 0.74; LA, 1.67	2016−2018	Supraclavicular BPB	VAS 0−10
Wang et al. 2025 [14]	Retrospective cohort (BPB vs. LA)	BPB, 60; LA, 60	BPB, 53.1 ± 9.9; LA, 54.2 ± 11.5	BPB, 1.14; LA, 1.00	2021−2023	Axillary BPB	VAS 0−10
Wen et al. 2025 [15]	RCT (SCNB vs. LA)	SCNB, 123; LA, 123	SCNB, 61 (54−68); LA, 63 (55−69)	SCNB, 1.12; LA, 1.24	2022−2023	SCNB (LACN + SRN; MCN as alternative)	VAS 0−10
Shimizu et al. 2025 [8]	Retrospective cohort (SCNB vs. LA)	SCNB, 168; LA, 50	SCNB, 73.3 ± 12.0; LA, 74.9 ± 11.2	SCNB, 1.90; LA, 1.50	2023−2023	SCNB (LACN ± SRN) combined with USIA	NRS 0−10

Abbreviations: BPB, brachial plexus block; IA, infiltration anesthesia; LA, local anesthesia; LACN, lateral antebrachial cutaneous nerve; M ± SD, mean ± standard deviation; Male/female ratio, the decimal values represent ratios rather than proportions; MCN, musculocutaneous nerve; Med (IQR), median (interquartile range); NRS/NRPS, numeric rating scale; PTA, percutaneous transluminal angioplasty; RCT, randomized controlled trial; SCNB, selective cutaneous nerve block; SRN, superficial radial nerve; UGNB, ultrasound‐guided nerve block; USIA, ultrasound‐guided infiltration anesthesia; VAS, visual analog scale.

### 3.3 Risk of Bias and Study Quality

Randomized trials were evaluated using the Cochrane RoB 2 tool, and observational studies using the NOS. Most domains were low to moderate risk; item‐level downgrades are shown in the ROB2 figure and/or NOS table, with no critical risks that would preclude quantitative synthesis.

### 3.4 Primary Outcome—Pain Intensity

**Figure 2:**
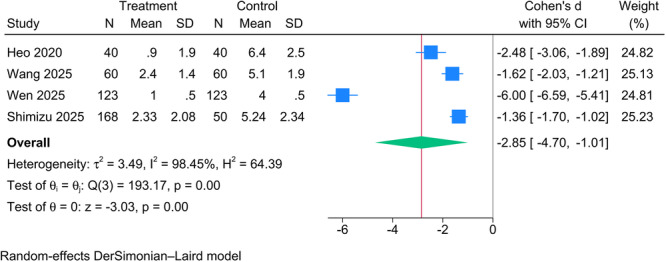
Pain forest plot of the included eligible studies. Pain scores measured using VAS/NRS were first harmonized to a 0–10 metric before effect‐size calculation, whereas pooled SMDs are unitless. Cohen's *d* was used as the SMD effect‐size metric in the forest plot. τ^2^, between‐study variance; CI, confidence interval; DL, DerSimonian–Laird; *I* ^2^, Higgins' I‐squared; LA, local anesthesia; NRS, numeric rating scale; SMD, standardized mean difference; UGNB, ultrasound‐guided nerve block; VAS, visual analog scale.

This was a prespecified primary outcome. Four eligible studies were included in the pain meta‐analysis. UGNB was associated with substantially lower intra‐procedural pain than LA in the random‐effects model (SMD −2.85, 95% CI −4.70 to −1.01; *p* < 0.001), although heterogeneity remained extreme (*I* ^2^ = 98.45%; Figure 2). All individual studies showed an effect direction favoring UGNB. The Galbraith plot identified Wen et al. as the most influential study, with a markedly larger standardized pain effect than the remaining studies; therefore, its influence was further assessed in leave‐one‐out sensitivity analyses rather than excluding it from the primary synthesis.

### 3.5 Sensitivity Analyses

**Figure 3:**
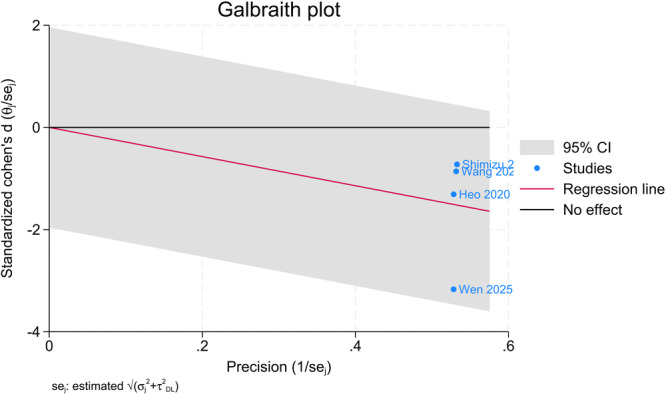
Galbraith plot for pain among the included eligible studies. Wen et al. was identified as the most influential study and was further assessed using leave‐one‐out sensitivity analysis rather than being removed from the primary synthesis. CI, confidence interval; DL, DerSimonian–Laird; SMD, standardized mean difference.

**Figure 4:**
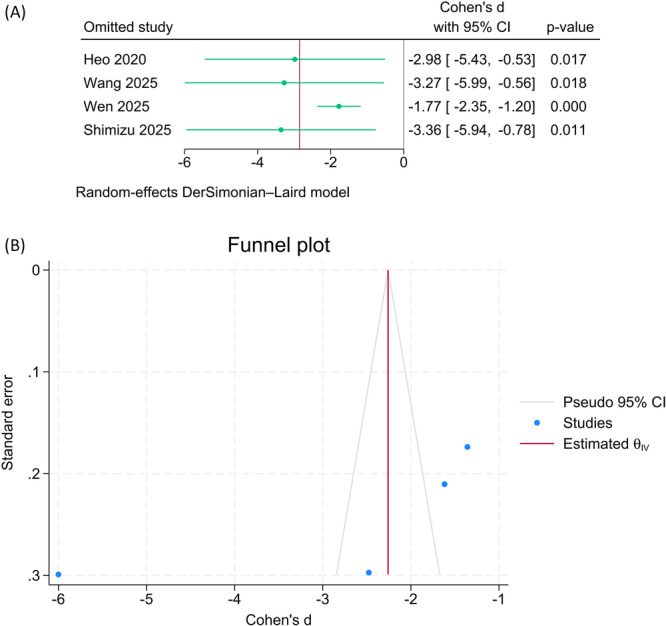
Sensitivity and small‐study effects among the included eligible studies. (A) Leave‐one‐out influence analysis for the pain outcome. (B) Exploratory funnel plot for the pain outcome. Cohen's *d* was used as the SMD effect‐size metric. Because fewer than 10 studies were included, the funnel plot should be interpreted as an exploratory visualization only. CI, confidence interval; DL, DerSimonian–Laird; SMD, standardized mean difference.

These were exploratory analyses. The Galbraith plot was used to visualize heterogeneity and identified Wen et al. as the most influential study (Figure 3). Leave‐one‐out influence analysis showed that omission of any single study did not change the direction or statistical significance of the pooled effect. Notably, after omitting Wen et al., the pooled effect remained significant and more stable (SMD −1.77, 95% CI −2.35 to −1.20; *p* < 0.001), supporting the robustness of the analgesic benefit of UGNB (Figure 4A).

### 3.6 Subgroup Analyses

**Figure 5:**
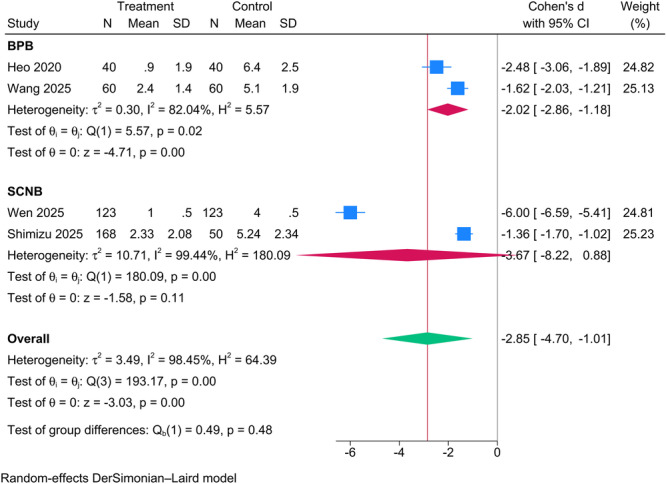
Subgroup meta‐analysis by UGNB subtype among the included eligible studies. The BPB subgroup included Heo et al. and Wang et al., whereas the SCNB subgroup included Wen et al. and Shimizu et al. Cohen's *d* was used as the SMD effect‐size metric. τ^2^, between‐study variance; BPB, brachial plexus block; CI, confidence interval; DL, DerSimonian–Laird; *I* ^2^, Higgins' I‐squared; LA, local anesthesia; SCNB, selective cutaneous nerve block; SMD, standardized mean difference; UGNB, ultrasound‐guided nerve block.

This subgroup comparison was prespecified in the study protocol (Figure 5). In the BPB subgroup, UGNB was associated with significantly lower pain than LA (SMD −2.02, 95% CI −2.86 to −1.18; *p* < 0.001; *I* ^2^ = 82.04%). In the SCNB subgroup, the pooled estimate also favored UGNB but had a very wide CI and extreme heterogeneity (SMD −3.67, 95% CI −8.22 to 0.88; *p* = 0.11; *I* ^2^ = 99.44%). No statistically significant difference was detected between BPB and SCNB subgroups (Qb = 0.49; *p* = 0.48). Therefore, the current evidence does not establish the superiority of one UGNB subtype over another.

An exploratory subgroup analysis by study design was also conducted using the four eligible studies. The analgesic benefit of UGNB over LA remained directionally consistent in both RCTs and retrospective cohort studies. Among RCTs, the pooled SMD was −4.24 (95% CI −7.69 to −0.79), whereas among retrospective cohort studies, the pooled SMD was −1.46 (95% CI −1.73 to −1.20). No statistically significant between‐subgroup difference was observed by study design (Qb = 2.47; *p* = 0.12; Figure S1). Because only two studies were available in each subgroup, this exploratory analysis should be interpreted cautiously.

### 3.7 Safety Outcomes

**Figure 6:**
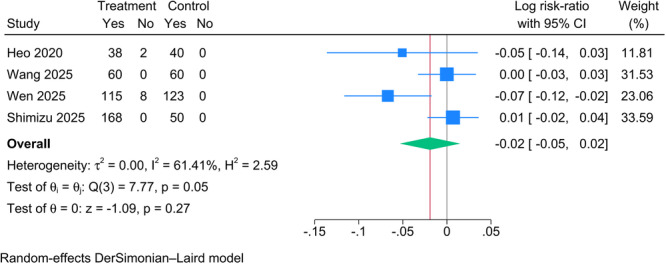
Safety analysis among the included eligible studies. Forest plot of the complication‐free outcome on the log risk‐ratio scale. “Yes” indicates no reported complication, whereas “No” indicates at least one reported complication. CI, confidence interval; LA, local anesthesia; RR, risk ratio; UGNB, ultrasound‐guided nerve block.

This was a prespecified safety analysis (Figure 6). Across the four eligible studies, no clinically meaningful difference in the complication‐free outcome was observed between UGNB and LA. The pooled log RR was −0.02 (95% CI −0.05 to 0.02; *p* = 0.27; *I* ^2^ = 61.41%), corresponding approximately to an RR of 0.98 (95% CI 0.95 to 1.02). No major nerve injury, pneumothorax, or persistent motor deficits were reported in any included study.

### 3.8 Publication Bias

Because fewer than 10 studies were included, formal assessment of publication bias or small‐study effects was considered underpowered and was not emphasized. A funnel plot was generated only for exploratory visualization (Figure 4B).

## 4 Discussion

In summary, this meta‐analysis of four eligible studies showed that UGNB was associated with substantially lower intra‐procedural pain than LA during angioplasty of dysfunctional AVFs. The direction of effect consistently favored UGNB across all included studies. Although heterogeneity remained extreme, Galbraith and leave‐one‐out analyses indicated that the overall conclusion was not driven solely by any single study. The safety analysis did not show a clinically meaningful difference between UGNB and LA, and no major nerve injury, pneumothorax, or persistent motor deficit was reported [8, 13, 14, 15].

The subgroup analysis by block type should be interpreted cautiously. BPB was associated with a significant reduction in pain versus LA, whereas the SCNB subgroup showed a directionally favorable but statistically imprecise pooled estimate with extreme heterogeneity. The between‐subgroup difference was not statistically significant. Therefore, current evidence supports UGNB as an analgesic strategy overall but does not establish a clear superiority of BPB over SCNB, or vice versa. Technique selection should therefore be individualized according to lesion site, the need for motor preservation, patient tolerance, and local operator expertise.

Regarding safety, the pooled analysis did not show a clinically meaningful difference between UGNB and LA. Because adverse events were infrequent and inconsistently defined, this safety result should be interpreted as reassuring but not definitive [8, 13, 14, 15]. Teams should still counsel patients about temporary motor weakness after BPB and use local protocols for block‐regression assessment before discharge.

From a pragmatic perspective, these results support graded, patient‐centered use rather than routine application in all patients. In centers with ultrasound‐guided regional anesthesia expertise, UGNB may be particularly useful for anxious or low‐tolerance patients, for recurrent stenoses requiring repeated PTA, or situations in which sedatives are undesirable because of frailty or cardiopulmonary risk [5]. Technique selection should balance analgesic requirements, preservation of motor function, same‐day discharge needs, and operator experience.

Between‐study variability likely arose from block type, local anesthetic choice and concentration, study design, operator and ultrasound experience, PTA lesion characteristics, and pain‐scale format [10]. Galbraith analysis identified Wen et al. as the most influential study, largely because of its exceptionally large standardized pain effect. However, leave‐one‐out analysis showed that the pooled effect remained statistically significant after omission of any single study, including Wen et al. Because only four studies were available, funnel plots were used only for exploratory visualization, and formal small‐study effect testing was not emphasized [11].

Finally, with respect to generalizability and future research, most participants were older adults with ESKD treated in Asian centers, reflecting an important but geographically limited evidence base [8, 13, 14, 15]. In this context, better analgesia without more safety concerns may improve cooperation, reduce sedative needs, and facilitate outpatient workflows [3, 5]. Multicenter randomized trials directly comparing BPB and SCNB, standardizing dose, mixture, and volume, measuring time to functional recovery, and evaluating patency and cost‐effectiveness are needed to refine technique selection across patient subgroups.

## 5 Conclusion

In this meta‐analysis of four eligible studies, UGNB was associated with substantially lower intra‐procedural pain than LA during AVF PTA, without a clear increase in adverse events. Current evidence does not establish a statistically significant difference between BPB and SCNB, and subgroup findings should be interpreted cautiously because of the small number of studies and substantial heterogeneity. UGNB may be considered in selected patients when improved procedural tolerance is prioritized or when sedation poses additional risk. Larger multicenter trials are required to compare UGNB subtypes directly and to evaluate functional recovery, access outcomes, and cost‐effectiveness.

## Author Contributions

**Wenshen Pu:** conceptualization, data curation, formal analysis, investigation, methodology, visualization, writing – original draft, writing – review and editing, project administration. **Zhihu Chen:** data curation, formal analysis, investigation, methodology, visualization, writing – original draft, writing – review and editing, project administration. **Zhangjian Xu:** data curation, investigation, software, visualization, writing – review and editing. **Yi Zhang:** data curation, investigation, resources, validation, writing – review and editing. **Zhidao Wang:** data curation, investigation, validation, resources, writing – review and editing. **Bingjie Wang:** conceptualization, methodology, supervision, project administration, resources, validation, writing – review and editing. **Ziming Wan:** conceptualization, methodology, supervision, validation, resources, writing – review and editing. All authors have read and approved the final version of the manuscript.

## Funding

The authors have nothing to report.

## Ethics Statement

Ethics approval was not required because this systematic review and meta‐analysis used only previously published aggregate data and did not involve individual patient recruitment or access to identifiable patient information.

## Conflicts of Interest

The authors declare no conflicts of interest.

## Transparency Statement

The manuscript guarantor, Ziming Wan, affirms that this manuscript is an honest, accurate, and transparent account of the study being reported; that no important aspects of the study have been omitted; and that any discrepancies from the study as planned and registered have been explained.

## Supporting information


**Figure S1:** Exploratory subgroup meta‐analysis by study design among the included eligible studies. The RCT subgroup included Heo et al. (2020) and Wen et al. (2025), whereas the retrospective cohort subgroup included Wang et al. (2025) and Shimizu et al. (2025). Pain scores measured using VAS/NRS were first harmonized to a 0–10 metric before effect‐size calculation; pooled SMDs are unitless. Cohen's d was used as the SMD effect‐size metric. Random‐effects DerSimonian–Laird models were used. Abbreviations: CI, confidence interval; LA, local anesthesia; RCT, randomized controlled trial; SD, standard deviation; SMD, standardized mean difference; UGNB, ultrasound‐guided nerve block

Supporting File

## Data Availability

The data that support the findings of this study are available from the corresponding author upon reasonable request. Ziming Wan had full access to all of the data in this study and takes complete responsibility for the integrity of the data and the accuracy of the data analysis.
